# Caries-Preventive Effect of High-Viscosity Glass Ionomer and Resin-Based Fissure Sealants on Permanent Teeth: A Systematic Review of Clinical Trials

**DOI:** 10.1371/journal.pone.0146512

**Published:** 2016-01-22

**Authors:** Steffen Mickenautsch, Veerasamy Yengopal

**Affiliations:** Systematic Review initiative for Evidence-based Minimum Intervention in Dentistry/Department of Community Dentistry, Faculty of Health Sciences, University of the Witwatersrand, Johannesburg, South Africa; Ottawa Hospital Research Institute, CANADA

## Abstract

**Background:**

Glass-ionomers are traditionally regarded to be inferior to resin as fissure sealants in protecting teeth from dental caries, due to their comparatively lower retention rate. Unlike low-viscosity glass-ionomers, high-viscosity glass-ionomer cements (HVGIC) are placed as sealants by pressing the material into pits and fissures with a petroleum-jelly-coated index finger. Hence, HVGIC sealants are assumed to penetrate pits and fissures deeper, resulting in a higher material retention rate, which may increase its caries-preventive effect.

**Methods:**

The aim of this review was to answer the question as to whether, in patients with fully erupted permanent molar teeth, HVGIC based fissure sealants are less effective to protect against dental carious lesions in occlusal pits and fissures than resin-based fissure sealants? A systematic literature search in eight databases was conducted. Heterogeneity of accepted trials and imprecision of the established evidence were assessed. Extracted sufficiently homogenous datasets were pooled by use of a random-effects meta-analysis. Internal trial validity was evaluated. The protocol of this systematic review was registered with the International Prospective Register of Systematic Reviews (PROSPERO / Nr.: CRD42015016007).

**Results:**

Seven clinical trials were provisionally included for further review. Of these, one was excluded. Seven trial reports reporting on six trials were accepted. From these, 11 datasets were extracted and pooled in four meta-analyses. The results suggest no statistically significant differences after up to 48 months and borderline significant differences in favour of HVGIC sealants after 60 months (RR 0.29; 95% CI: 0.09–0.95; p = 0.04 / RD -0.07; 95% CI: -0.14, -0.01). The point estimates and upper confidence levels after 24, 36, 48 and 60 months of RR 1.36; RR 0.90; RR 0.62; RR 0.29 and 2.78; 1.67; 1.21; 0.95, respectively, further suggest a chronological trend in favour of HVGIC above resin-based sealants. The internal trial validity was judged to be low and the bias risk high for all trials. Imprecision of results was considered too high for clinical guidance.

**Conclusion:**

It can be concluded that: (i) Inferiority claims against HVGIC in comparison to resin-based sealants as current gold-standard are not supported by the clinical evidence; (ii) The clinical evidence suggests similar caries-preventive efficacy of HVGIC and resin-based sealants after a period of 48 months in permanent molar teeth but remains challenged by high bias risk; (iii) Evidence concerning a possible superiority of HVGIC above resin-based sealants after 60 months is poor (even if the high bias risk is disregarded) due to imprecision and requires corroboration through future research.

## Introduction

Several reports have established the clinical efficacy [1–3] and cost-effectiveness [4,5] of sealants in reducing carious lesions in occlusion pits and fissures of molar teeth. Traditionally, resin composite has been placed as the most commonly used sealant material [6–9]. The effect of this material relies on its micro-retention, due to created enamel tags after acid etching. However, resin composite is moisture-sensitive and under wet conditions Glass Ionomer Cement (GIC) may be used as an alternative based on its hydrophilic characteristics [10].

Yengopal et al. conducted a systematic review of clinical trials with meta-analysis (cut-off search date: 15 January 2008) in order to appraise the clinical evidence regarding the caries-preventive effect of GIC sealants in comparison to resin composite. Its result showed that neither material was superior the other for the outcomes investigated [11]. Three years later, this systematic review was updated with its outcome being in agreement with that of the original systematic review [12] and another study established in April/May 2012 that the conclusions of the systematic review were still current [13].

In the previous systematic reviews and its subsequent updates no distinction was made between low- and high-viscosity GIC (HVGIC) for use as fissure sealant when compared to resin-based sealants (the current gold standard). In the past, HVGIC (as opposed to the most commonly studied low-viscosity GICs) have been applied as sealant material within the context of the atraumatic restorative treatment (ART) approach [14]. Initial observations showed a higher retention rate than low-viscosity GIC based sealants, particularly when placed using the press-finger technique. In addition, low carious lesion development on HVGIC sealed teeth and no operator effect were reported [14].

Defining HVGICs based on the material’s powder/liquid ratio or compressive strength has proven to be difficult because *in-vitro* findings using these variables have shown conflicting results [15]. However, a clinical distinction between low and high-viscosity conventional GICs, has been possible, as published studies have shown that high-viscosity GICs, when used as tooth restorations (such as Ketac Molar and Fuji IX), appear to have similar clinical merit as amalgam, whilst low-viscosity GICs were shown to be clearly inferior [16]. HVGICs when used as sealants are ideally placed following the recommendations by Frencken et al., 1996 in line with the atraumatic restorative treatment (ART) approach [17].

The caries preventive effect of GICs has been ascribed to its adhesion due to calcium bonds [18] and its ability to leach fluoride into the oral cavity [19]. As GIC sealants fracture cohesively some parts may remain deep in the pits and fissures and thus may continue to offer dental caries prevention. In addition to these characteristics, common to both, low- and high-viscosity GICs, HVGICs when placed using the press-finger technique, may penetrate pits and fissures deeper, resulting in a higher material retention rate compared to low-viscosity GICs which thus may further contribute to its superior caries-preventive effect in molar teeth.

In contrast to past systematic review findings [11–13], GICs are traditionally regarded as inferior to resin as fissure sealants. Simonsen stated in 2002 that glass-ionomer sealants have failed “miserably” in comparison to resin-based sealants, showing very poor retention and added that even if they inhibit caries for longer time periods, this would not compensate for the poor retentive properties of the material [8]. Locker et al. (2003) concurred that while auto-polymerizing sealants and visible light curing sealants have high retention rates, GICs have lower retention rates [1] and Kühnisch et al. concluded in 2012 that because of their lower retention rates than conventional resin-based sealants, GIC’s cannot be recommended for routine clinical use in dental practice [9].

This systematic review seeks to answer the PICO question (representing: Patient, Intervention, Control intervention and clinical measured Outcome) as to whether, in patients with fully erupted permanent molar teeth, high-viscosity glass-ionomer based fissure sealants are less effective to protect against dental carious lesions in occlusal dental pits and fissures than resin-based fissure sealants.

## Methods

The protocol of this systematic review has been registered with the International Prospective Register of Systematic Reviews (PROSPERO / Nr.: CRD42015016007) and was published in an open access journal [20].

### Systematic Literature Search

Both authors searched the following electronic databases independently: (1) General international databases: CENTRAL accessed via Cochrane Library, MEDLINE accessed via PubMed; (2) Open access sources: Biomed Central, Database of Open Access Journals (DOAJ); (3) Regional databases: [a] Africa: Sabinet, [b] India: IndMed; (4) Grey-Literature sources: OpenSIGLE, Google Scholar. Reference check of all included trial reports, as well as additional journal hand searching was conducted. The details of the search strategy, including search terms and search dates per database are presented in Section A in S1 File. Citations were eligible for possible inclusion if in line with the following criteria:

1. Clinical trials (trials on animals, in-situ, in-vitro trials not included);
2. Controlled trials: including control- and test group(s) (1-arm longitudinal trials not included);
3. Trial focus relevant to review question;
4. Prospective trials (retrospective trials not included);
5. Full trial reports (abstracts without full reports not included);
6. Follow-up period minimum 24 months;
7. Treatment on fully erupted caries-free molar teeth in the permanent dentition;
8. High-viscosity glass-ionomers as test intervention;
9. Resin-based materials as control intervention.

Trial participants included all patients of any age, gender or place of origin.

Articles were further excluded according to the criteria:

1. No computable data reported;
2. Test and control groups not evaluated the same way;
3. Low-viscosity chemically cured, resin-modified or light-cured glass-ionomers as test intervention;
4. Trials published in any other language than English.

Titles and abstracts of identified citations from data sources were scanned by the two authors in duplication, for possible inclusion in line with the inclusion criteria. Articles with a suitable title but without listed abstract were retrieved in full copy. All included articles were judged separately by authors for possible exclusion against criteria agreed upon in the study protocol. Disagreements between authors were resolved through discussion and consensus.

### Data Collection from Accepted Trials and Analysis

The two authors extracted data from accepted trials independently without being blinded to authors, institutions, journal name, as well as trial results. Disagreements between authors concerning data extracted were solved through discussion and consensus. All data were entered in specifically designed data sheets in MS Excel. The following data were extracted:

1. General important information: Article reference; place of trial; age, gender of trial participants; selection criteria; baseline caries experience; fluoride exposure; type of study design; information on trial operators, evaluators and clinical settings; information on caries diagnostic criteria; failure criteria; caries assessment method.
2. Information per test- and control group: Product name of sealant material used; type of molar tooth sealed: 1^st^/2^nd^ /3^rd^ Molar; upper/lower jaw; left/right side; number of participants at beginning of trial (BSL); assessment method used (clinical examination, X-Ray, etc); dental caries assessment criteria followed; follow-up period (in months); number of evaluated units at end of follow-up period (N); number of failures (n) for dichotomous data.
3. Information for test group: Press-finger technique used during glass-ionomer placement (yes / no).
4. Information for control group: Fluoride containing (yes / no); information on sealant placement procedure related to enamel etching and moisture control.
5. Verbatim quotes relevant to selection-, performance- and detection bias risk: Selection bias: Random sequence generation, concealment of the sequence allocation; Performance bias: Operator blinding; patient blinding; Detection bias: Evaluator blinding; Unit of randomization; Unit of statistical analysis.

The outcome measure was the number of teeth that have developed carious lesions on previously sealed occlusal pits and fissures (n = Number of failures) from the total number of evaluated teeth (N). A dataset was defined as any extracted set of n / N for test- and control group. For each dataset the Risk ratio (RR) with 95% Confidence intervals (CI) and p-values were computed. Statistical significance was set at alpha 5%. For computation of all effect estimates (with 95% CI) the statistical software programme RevMan 4.2 was used.

In order to fulfill criteria for clinical and methodological homogeneity, datasets from the accepted trials did not differ in the following minimum set of characteristics: Length of follow-up period; type of sealant application per intervention group; applied caries assessment criteria; type of sealed molar tooth; age of patients; for control group: fluoride containing (yes / no); for test group: use of press-finger technique (yes / no).

The I^2^ –test with 95% CI was used to establish whether any statistical heterogeneity existed between datasets that were assumed to be sufficiently clinically and methodologically homogenous. Thresholds for I^2^ point estimates (in %) and its upper confidence values were used in order to interpret the test results [21]: 0–40% = might not be important; 30–60% = may represent moderate heterogeneity; 50–90% = may represent substantial heterogeneity; 75–100% = considerable heterogeneity. For computation of all I^2^ point estimates with 95% CI the software programme MIX 1.7 was used [22]. The 95% Confidence intervals of the I^2^ point estimates were used for interpretation.

Identified (clinically/methodologically) homogenous datasets with a measured I^2^ point estimate not exceeding 60% for statistical heterogeneity were pooled using random-effects meta-analysis with RevMan 4.2 software. A pooled Risk ratio (RR with 95% CI) was computed. In addition, any pooled effect estimate, indicating statistical significance (p < 0.05), was also computed as an absolute outcome measure (Risk difference—RD) with 95% Confidence intervals (CI) and p-values, as well as an illustrative comparative risk, i.e. the number of failures out of 100, for both test- and control intervention was generated with help of the Visual Rx—Statin Calculator by Cates [23,24]. Statistical significance was set at alpha 5% for all meta-analysis results.

### Imprecision Assessment

In line with GRADE recommendation [25] the level of imprecision of the appraised evidence was assessed. In order to assess whether included data had sufficient statistical power for the detection of meaningful differences between the compared interventions, post hoc analysis of sample size sufficiency was conducted for each conducted meta-analysis. The analysis was based on the following assumptions:

- Risk of type I error (risk of falsely detecting a difference), α = 5%
- Risk of type II error (risk of not detecting a true difference), β = 20%
- Power to detect a 10 percentage points difference (in line with Liu et al., 2014) [26]

Analysis was conducted using the formula by Pocock, 1983 [27] for calculating the required sample size (N_R_): N_R_ = [p_1_(100—p_1_) + p_2_(100—p_2_) / (p_2_—p_1_)^2^] ƒ(α, β), with:

1. p1 = p2 + 10 (assumed test group event rate)
2. p2 = Control group event rate n2/N2
3. $${{\text{(p}}_{\text{2}}{\text{ - p}}_{\text{1}}\text{)}}^{\text{2}}{\text{ = 10}}^{\text{2}}\text{ = 100}$$
4. ƒ(α, β) = ƒ(0.05, 0.20) = 7.9

In line with GRADE guidelines [25], the calculated required sample size was considered as the optimal information size (OIS) against which the total number of analyzed units per meta-analysis was compared (N_T_). If the latter was lower than the calculated OIS then imprecision of the established evidence was assumed. Furthermore, an imprecision threshold of 0.5% (Risk difference) [25] was considered for assessing the confidence intervals of the pooled results.

### Assessment of Bias Risk

Selection-, detection- and performance bias risk was assessed using the set of criteria presented in Section B in S1 File. Both authors conducted the assessment independently. Disagreements were resolved by discussion and consensus.

In order to assess attrition bias risk, a worst- and best-case scenario was assumed. Both, worst- and best-case scenario, provide the minimum and maximum outcome value beyond which neither lower nor higher values are possible. Both scenario values have the same probability to correspond with the true intervention outcome as any other possible scenario in between these extremes. Both values were calculated when the number of lost trial participants per intervention group was reported in the trial reports. The results were then compared to the intervention outcomes computed for participants available to follow-up and on this basis conclusions concerning attrition bias risk were drawn: i.e. high risk of attrition bias was assumed, if the computed outcomes between worst- and best-case scenario and the intervention outcomes computed for participants available to follow-up differed significantly.

The worst-case scenario was calculated by adding the number of lost-to-follow-up participants in the test group to the failures of that group and adding the number of lost-to-follow-up participants in the control group to the successes of that group. The best-case scenario was calculated by adding the number of lost-to-follow-up participants in the test group to the successes of that group and adding the number of lost-to-follow-up participants in the control group to the failures of that group. The method to assess attrition bias risk as sensitivity analysis by calculation of best/worse case scenarios was developed in collaboration with the School of Statistics & Actuarial Science, University of the Witwatersrand and applied in a number of published systematic reviews [12,28,29].

### Assessment of Publication Bias Risk

It was planned to compute the I^2^ point-estimate with 95% CI of all extracted datasets. High statistical in-between-datasets heterogeneity as per thresholds [21] would have been taken under consideration when assessing publication bias risk by graphical and statistical methods, such as funnel plot and Egger’s regression. Assessment of publication bias risk was not planned if the number of extracted datasets was < 10.

## Results

### Systematic Literature Search and Data Extraction

**Fig 1:**
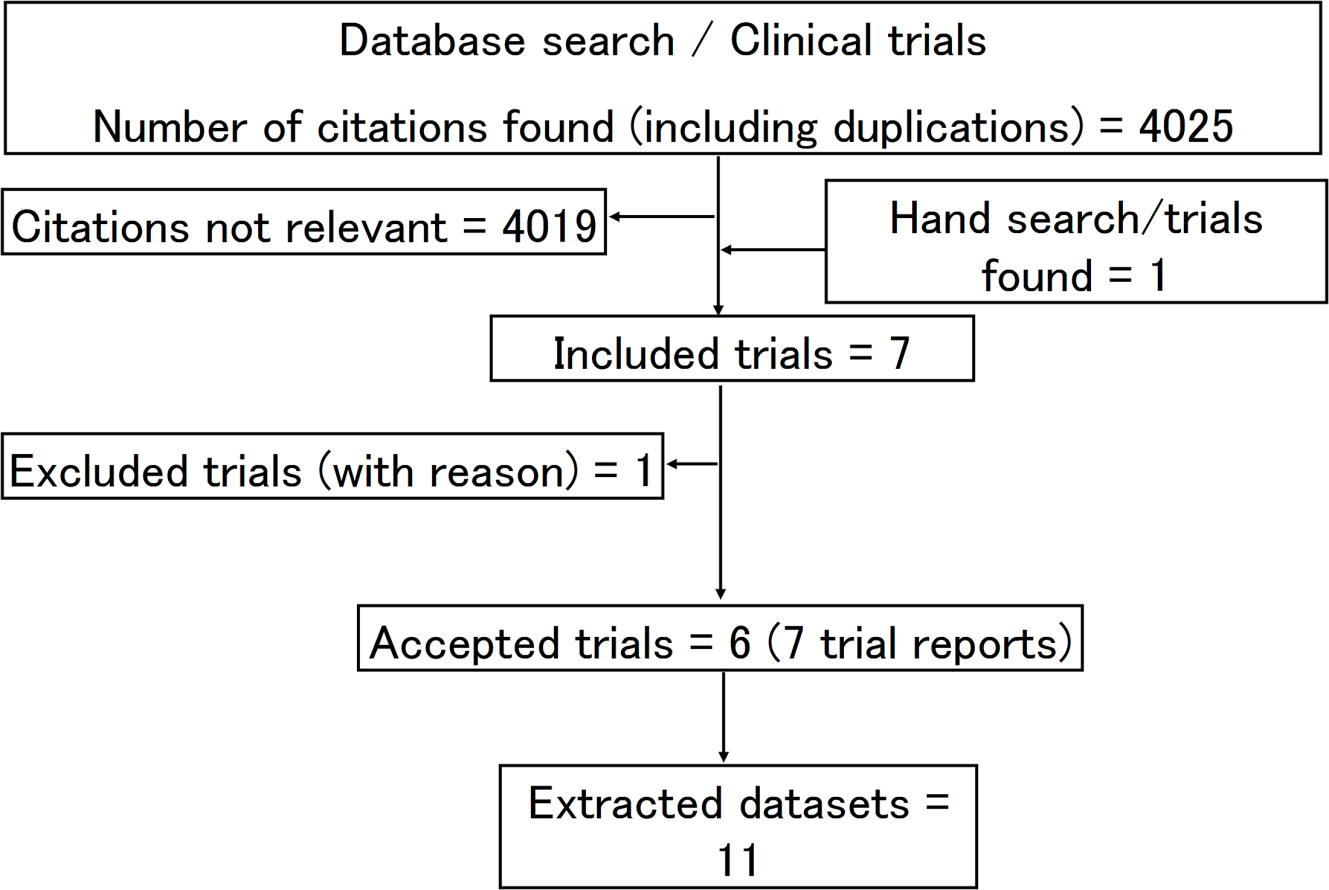
Flow diagram of trial selection.

Fig 1 provides information on the number of citations identified. From the 4025 found citations, seven clinical trials [14, 26,30–35] were provisionally included for further review. Of these, one trial [34] did not report carious lesion development on sealed teeth as a measured outcome and was thus excluded. Six trials were finally accepted for data extraction [14,26,30–33,35]. Of these, two separate reports were published for one trial, one for a 24-month follow-up period [32] the other for a 48-month follow-up period [33].

From the seven accepted trial reports, 11 datasets (DS 01–11) were extracted. The datasets and extracted general information, as well as verbatim quotes related to internal trial validity are presented per trial in Tables 1–3 and Section B in S2 File. Two trials were conducted in China [25,32,33], two in Brazil [29,35], one each in Syria [14] and Turkey [31]. The age of trial subjects where similar in all trials with a mean age of 7.8 years [14,26,32,33], an age range between 5–8 years [30], 7–11 years [31] and 6–7 years [35]. Only three of the seven trial reports included information about potential fluoride exposure (from tooth paste [26,30] and water fluoridation [35]). All trials placed sealants of first permanent molars, only. However, no trial report included information about the location of the sealed molar teeth in the oral cavity. The press-finger technique was applied in all trials for the placement of HVGIC in the test group. The HVGIC material was Fuji IX in two trials [14,30], Ketac Molar Easymix in three trials [26,32,33,35] and Ketac Molar in one trial [31]. Four trials placed fluoride containing resin-based material in the control group [26,31–33,35], while two trials placed resin materials without fluoride [14,30]. Four trials [14,30,32,33,35] followed a parallel group and two trials a split mouth study design [26,31]. The maximum follow-up periods of the accepted trial reports were 60 months [14,30], 48 months [33], 36 months [31,35] and 24 months [26,32].

**Table 1:** General trial information–Trial characteristics.

Trial	DS	Place of trial	Study design	Follow-up Period (in months)	Operator type	Help by Assistant	Age	Gender	Baseline caries experience	Fluoride Exposure	Patient selection Criteria (verbatim)	Tooth selection Criteria (verbatim)
Beiruti et al. [14]	1	Syria^1^	PG	24	Oral hygienist	No	Mean 7.8 years	46 boys, 57 girls	Not reported	Not reported	Not reported	“(1) sound pits and fissures in fully erupted first molars; (2) pits and fissures diagnosed with an early enamel lesion (score 1) and /or small dentine lesion (score 2)”
Beiruti et al. [14]	2	Syria^1^	PG	36	Oral hygienist	No	Mean 7.8 years	46 boys, 57 girls	Not reported	Not reported	Not reported	(See above)
Beiruti et al. [14]	3	Syria^1^	PG	48	Oral hygienist	No	Mean 7.8 years	46 boys, 57 girls	Not reported	Not reported	Not reported	(See above)
Beiruti et al. [14]	4	Syria^1^	PG	60	Oral hygienist	No	Mean 7.8 years	46 boys, 57 girls	Not reported	Not reported	Not reported	(See above)
Barja- Fidalgo et al. [30]	5	Brazil^2^	PG	60	Graduate students	No	5(6?) to 8 years / Mean age 6.8 years (SD +/- 0.98)	14 boys, 22 girls	dmfs Test group: 16.5 (95% CI: 10.60–22.40); Control group: 13.3 (95% CI: 8.50–8.10)	Tooth paste	“with at least 1 permanent first molar erupted and 2 or more primary molars decayed, filled, or extracted due to caries,”	“All the permanent first molars that presented a sound occlusal surface or occlusal cariesat the D1 level (noncavitated enamel lesion)”
Oba et al. [31]	6	Turkey^3^	SM	36	Dentists	No	7 to 11 years	Not reported	Not reported	Not reported	Not reported	“(1) sound pits and fissures in fully erupted first molars; and (2) pits and fissures diagnosed with an early enamel lesion.”
Chen et al. [32]	7	China^4^	PG	24	Dentists	Not reported	Mean 8 years	Not reported	d2mft Test group: 6.2 (2.8); Control group: 6.4 (2.7)	Not reported	“dmft≥2”	“a fully erupted first permanent molar, no dentin caries lesion in pits and fissures of these molars, deep and/or intermediate pits or fissures, “
Zhang et al. [33]	8	Same study as: Trial by Chen et al. [32]		48	Zhang et al. [33]							
Liu et al. [26]	9	China^5^	Partial SM	24	Dentists	Yes	Mean 7.8 years	44% boys	DMFT: 0.54	(Fluoride Tooth Paste common on the market)?	“Children who did not have any major general health problems”	“permanent first molars with occlusal fissures which were deep (base of fissure cannot be seen) or presented with signs of incipient caries (opacity and discoloration seen when viewed wet), similar to ICDAS code 2”
Hilgert et al. [35]	10	Brazil^6^	PG	24	Pedodontists	Yes	6–7 years	126 boys, 116 girls	D2MFT Test group: 3.00; Control group: 3.37	Flouridated water	“good general health; 2) at least 2 cavitated dentine carious lesions in vital pain-free primary molars, assessed according to the second digit of the ICDAS II”	"erupted first permanent molars, with the occlusal surface fully visible and accessible; 4) high–caries risk occlusal surfaces in first permanent molars, determined by ICDAS II codes 2 and 3 or by a combination of ICDAS II code 1 and medium or deep fissures (assessed according to Symons et al. 1996); and 5) a signed consent form."
Hilgert et al. [35]	11	Brazil^6^	PG	36	Pedodontists	Yes	6–7 years	126 boys, 116 girls	D2MFT Test group: 3.00; Control group: 3.37	Flouridated water	(See above)	(See above)

DS = Dataset number; PG = Parallel group; SM = Split-mouth; CI = Confidence interval; ART = Atraumatic restorative treatment; SD = Standard deviation.

^1^ Damascus Clinical department, WHO Center;

^2^ Rio de J. Department of Paediatric Dentistry;

^3^ Kirikkale Portable equipment at schools;

^4^ Hubei (Wuhan) / Portable equipment at schools;

^5^ Shenzhen Portable equipment at schools;

^6^ Primary schools of Paranoá, a deprived suburban area of Brasilia.

**Table 2:** General trial information–Trial data.

Trial	DSNr	HVGIC (Test) group										Resin (Control) group													
		Sealant Material	PFT	Molar tooth			Unit of analysis					Sealant Material	F	ET	Removal of Etching gel	AD	MC	Molar tooth			Unit of analysis				
				Type	Jaw	Site	Type	BSL	n	N	LTF							Type	Jaw	Site	Type	BSL	n	N	LTF
Beiruti et al. [14]	1	Fuji IX GC	Yes	1st	Not reported	Not reported	Tooth	180	0	154	26	Visio-Seal ESPE	No	30 sec	Water rinsing	Yes	By suction	1st	Not reported	Not reported	Tooth	180	5	161	19
Beiruti et al. [14]	2	Fuji IX GC	Yes	1st	Not reported	Not reported	Tooth	180	3	154	26	Visio-Seal ESPE	No	30 sec	Water rinsing	Yes	By suction	1st	Not reported	Not reported	Tooth	180	7	138	42
Beiruti et al. [14]	3	Fuji IX GC	Yes	1st	Not reported	Not reported	Tooth	180	4	143	37	Visio-Seal ESPE	No	30 sec	Water rinsing	Yes	By suction	1st	Not reported	Not reported	Tooth	180	8	123	57
Beiruti et al. [14]	4	Fuji IX GC	Yes	1st	Not reported	Not reported	Tooth	180	1	80	100	Visio-Seal ESPE	No	30 sec	Water rinsing	Yes	By suction	1st	Not reported	Not reported	Tooth	180	6	76	104
Barja-Fidalgo et al. [30]	5	Fuji IX GC	Yes	1st	Not reported	Not reported	Tooth	46	2	21	25	Delton Dentsply	No	30 sec	Water rinsing	Yes	Cotton rolls	1st	Not reported	Not reported	Tooth	46	7	28	18
Oba et al. [31]	6	Ketac Molar 3MESPE	Yes	1st	Not reported	Not reported	Tooth	91	6	56	35	Fissurit F Voco	Yes	20 sec	Water rinsing	Yes	Cotton rolls	1st	Not reported	Not reported	Tooth	116	8	81	35
Chen et al. [32]	7	Ketac Molar Easymix 3MESPE	Yes	1st	Not reported	Not reported	Tooth	450	7	415	35	Ketac Molar Easymix/3MESPE	Yes	20 sec	Water rinsing	Yes	By suction	1st	Not reported	Not reported	Tooth	478	5	452	26
Zhang et al. [33]	8	Same study as: Trial by Chen et al. [32]						450	9	345	105											478	14	396	82
Liu et al. [26]	9	Ketac Molar Easymix 3MESPE	Yes	1st	Not reported	Not reported	Tooth	194	13	179	15	Clinpro 3MESPE	Yes	15–20 sec	Water rinsing	Yes	Cotton rolls	1st	Not reported	Not reported	Tooth	189	7	178	11
Hilgert et al. [35]	10	Ketac Molar Easymix 3MESPE	Yes	1st	Not reported	Not reported	Tooth	69	4	60	9	Fluoro-shield Dentsply	Yes	30 sec	Water rinsing	Yes	Cotton rolls	1st	Not reported	Not reported	Tooth	169	7	143	26
Hilgert et al. [35]	11	Ketac Molar Easymix 3MESPE	Yes	1st	Not reported	Not reported	Tooth	69	6	51	18	Fluoro-shield Dentsply	Yes	30 sec	Water rinsing	Yes	Cotton rolls	1st	Not reported	Not reported	Tooth	169	12	120	49

DS = Dataset number; BSL = Number of included sealants at baseline; n = Number of failed sealants; N = Number of evaluated sealants; LTF = Sealants lost to follow-up; PFT = Press finger technique used; F = Material including fluoride; ET = Etching time; MC = Moisture control; AD = Air drying; sec = Seconds.

**Table 3:** General trial information–Applied evaluation methods in trials.

Trial	Evaluators	Caries diagnostic criteria	Failure criteria	Assessment method
Beiruti et al. [14]	Calibrated, experienced evaluators (presumably dentists)	As per Beiruti et al., 2006 [14]	Dentinal Lesion; Restoration / Missing tooth due to caries	Clinical examination
Barja-Fidalgo et al. [30]	Calibrated examiner (presumably dentist)	Not reported	Dentinal Lesion or radiolucency in dentin (X-Ray)	Clinical and radiological examination
Oba et al. [31]	Not reported (presumably article authors)	Not reported	Caries present	Clinical examination
Chen et al. [32]	Calibrated, trained independent examiner (presumably dentist)	ART caries criteria	Dentinal lesion	Clinical examination
Zhang et al. [33]	Same study as: Trial by Chen et al. [32]			
Liu et al. [26]	Calibrated dentists	ICDAS	Dentinal lesion	Clinical examination
Hilgert et al. [35]	Independent dentists	ICDAS	Cavitated dentine carious lesions	Clinical examination

ICDAS = International Caries Detection and Assessment System; ART = Atraumatic restorative treatment.

### Data Analysis

The computed results of the extracted 11 datasets are presented in Section A in S2 File and indicate no statistically significant differences in the caries-preventive effect of both types of sealants.

**Fig 2:**
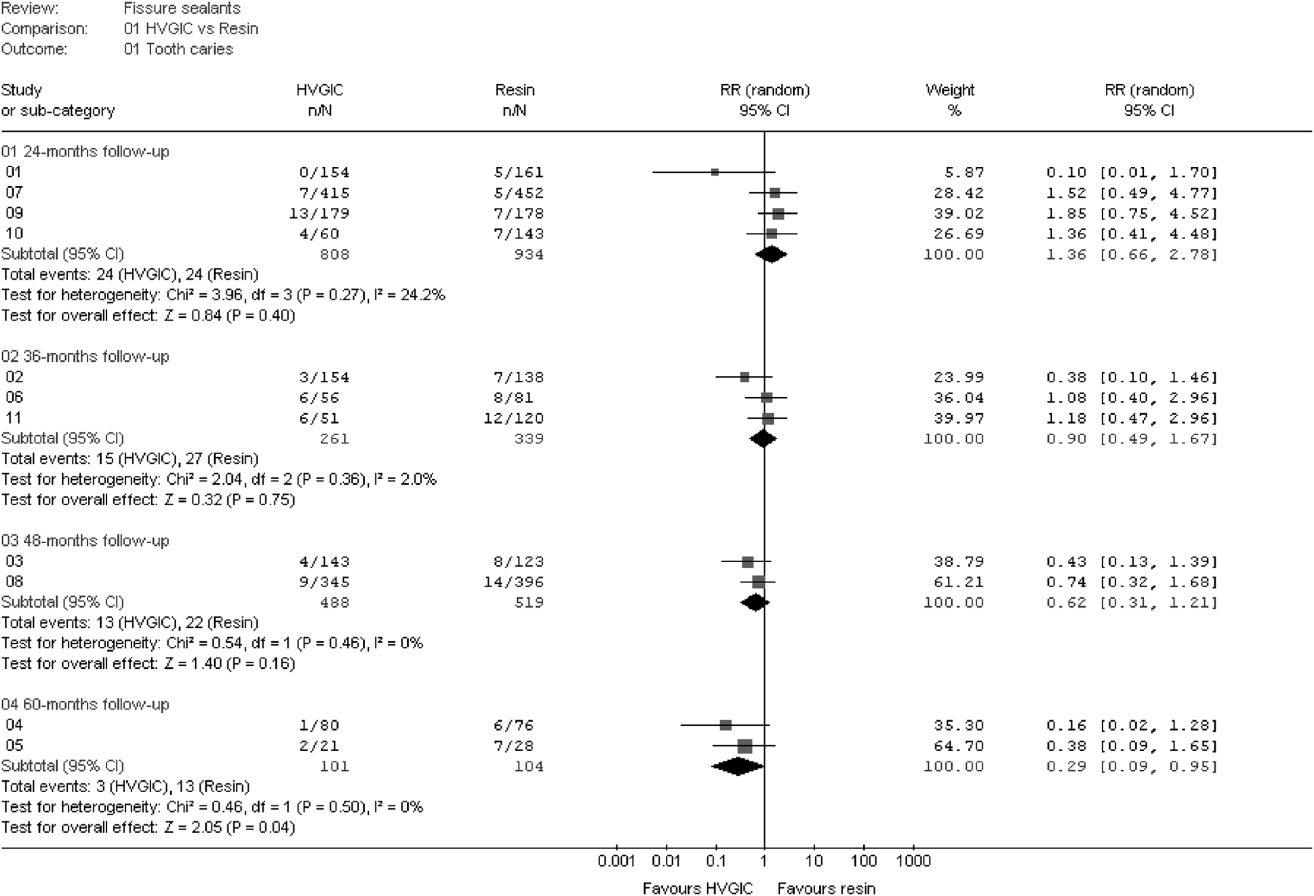
Meta-analysis results. HVGIC = High viscosity glass-ionomer cement; n = Number of sealed teeth with caries (events); N = Number of evaluated teeth; RR = Relative risk; CI = 95% Confidence interval. Number of datasets (DS) extracted from trials: 01–04 = Beiruti et al.; 05 = Barja-Fidalgo et al.; 06 = Oba et al.; 07 = Chen et al.*; 08 = Zhang et al.*; 09 = Liu et al.; 10,11 = Hilgert et al. *Reports of different follow-up periods from same trial.

Clinical and methodological inter-dataset heterogeneity was investigated. The details of established dataset characteristics are presented in Table 2 and Section C in S2 File. Based on the results, the 11 datasets were pooled in four meta-analyses (Fig 2). Complete clinical homogeneity was not achieved for all meta-analyses. Differences remained in the type of product brand of the sealant materials and fluoride content of the resin-based sealant material placed in the control groups. However, despite these remaining differences, statistical inter-dataset heterogeneity appeared moderate in line with the I^2^—point estimates: I^2^ = 24.2%, 95% CI: 0–88.2% (Meta-analysis 1); I^2^ = 2.0%, 95%CI: 0–89.8% (Meta-analysis 2); I^2^ = 0% (Meta-analysis 3); I^2^ = 0% (Meta-analysis 4). The upper confidence values for Meta-analysis 1 and 2 were observed to be high, indicating “substantial” heterogeneity (I^2^ = 88.2 and 89.8%, respectively), while the lower confidence levels were both at zero, thus presenting extremely wide confidence intervals. No confidence intervals could be computed for meta-analyses 3 and 4 as both included two datasets, only.

On basis of the moderate heterogeneity risk, four meta-analyses were computed for the 24-, 36-, 48- and 60-month follow-up periods (Fig 2). The meta-analyses results suggest no statistically significant differences after 24, 36 and 48 months and borderline significant differences in favour of HVGIC sealants after 60 months (RR 0.29; 95% CI: 0.09–0.95; p = 0.04). When the latter result was converted into the absolute measure of risk reduction (RD) it was found that placing HVGIC instead of resin-based sealants reduces the dental caries risk in the sealed pits and fissures by 7 percentage points (RD -0.07; 95% CI: -0.14, -0.95) after 60 months. In addition, the RD results were converted into the illustrative risk of 4 teeth with dental caries out of 100 HVGIC sealed teeth versus 13 teeth with dental caries out of 100 resin sealed teeth.

The point estimates and upper confidence levels after 24, 36, 48 and 60 months of RR 1.36; RR 0.90; RR 0.62; RR 0.29 and 2.78; 1.67; 1.21; 0.95, respectively, further suggest a chronological trend in favour of HVGIC above resin-based sealants (Fig 2).

Pooling of the data increased statistical power sufficiently (Section A in S2 File). However, none of the results exceeded the threshold of 0.5% (Risk difference), thus insufficient precision of the established evidence for clinical guidance was assumed.

### Assessment of Internal Trial Validity/Bias Risk

Assessment of selection- and performance-/detection bias risk was based on verbatim quotes extracted from all accepted trials (Section B in S2 File). The assessment results are presented in Table 4. On this basis, the internal trial validity was judged to be low and the bias risk high for all trials. Only one trial reported (published as two trial reports with varying follow up periods) the use of adequate methods for random sequence generation and concealment of the random sequence in order to prevent direct observation [32,33] and one trial reported the use of adequate methods for random sequence generation [30]. The unit of randomisation and the unit of reported statistical analysis was not the same in five trial reports [14,30,32,33,35]. Of these, three trials reported the patient [14,30,32,33] and one trial reported the school as the unit of randomisation [35] while using the sealed tooth as the unit of analysis. One trial that was reported in two trial reports [32,33] applied some form of statistical correction against data clustering. Two trials [26,31] reported the sealed teeth as unit of randomisation and analysis. None of the trials reported adequate methods of clinical operator masking as to the type of sealant placed (high performance bias risk) or adequate methods for masking evaluators during trial assessment (high detection bias risk). In addition, high attrition bias risk was identified for all trials (Tables 5 and 6).

**Table 4:** Assessment of Selection-, Detection/Performance bias risk.

Trial	Selection bias risk	Detection and performance bias risk		
		Operators	Patients	Evaluators
Beiruti et al. [14]	0	0	0	0
Barja-Fidalgo et al. [30]	D	0	0	0
Oba et al. [31]	0	0	0	0
Chen et al. [32]	C	0	0	0
Zhang et al. [33]	C	0	0	0
Liu et al. [26]	0	0	0	0
Hilgert et al. [35]	0	0	B	0

Selection bias risk:

Score 0 = No Adequate random sequence generation method reported / No method reported for concealing the random sequence allocation that may prevent its direct observation and its correct prediction

Score D = No method reported for concealing the random sequence allocation that may prevent its direct observation and its correct prediction

Score C = No method reported for concealing the random sequence allocation that may prevent its correct prediction

Detection and performance bias risk:

Score 0 = No adequate method reported for masking/blinding of patients and clinicians and evaluators

Score B = No evidence that shows masking/blinding was successful throughout the trial

**Table 5:** Assessment of attrition bias risk–Worst-case scenario.

Trial	DS	HVGIC group			Resin group			LTF adjusted effect estimate			Original effect estimate			Bias risk
		LTF	N = BSL teeth	n+ LTF	LTF	N+LTF	n	RR	95% CI	P	RR	95% CI	P	
Beiruti et al. [14]	01	26	180	26	19	180	5	5.20	2.04–13.24	0.0005**	0.10	0.01–1.70	0.11	Yes
Beiruti et al. [14]	02	26	180	29	42	180	7	4.14	1.86–9.21	0.0005**	0.38	0.10–1.16	0.16	Yes
Beiruti et al. [14]	03	37	180	41	57	180	8	5.13	2.47–10.62	<0.0001**	0.43	0.13–1.39	0.16	Yes
Beiruti et al. [14]	04	100	180	101	104	180	6	16.83	7.58–37.36	<0.00001**	0.16	0.02–1.28	0.08	Yes
Barja-Fidalgo et al. [30]	05	25	46	27	18	46	7	3.86	1.87–7.96	0.0003**	0.38	0.09–1.65	0.20	Yes
Oba et al. [31]	06	35	91	41	35	116	8	6.53	3.22–13.24	<0.00001**	1.08	0.40–2.96	0.87	Yes
Chen et al. [32]	07	35	450	42	26	478	5	8.92	3.56–22.35	<0.00001**	1.52	0.49–4.77	0.47	Yes
Zhang et al. [33]	08	105	450	114	82	478	14	8.65	5.04–14.84	<0.00001**	0.74	0.32–1.68	0.47	Yes
Liu et al. [26]	09	15	194	28	11	189	7	3.90	1.74–8.70	0.0009**	1.84	0.75–4.52	0.18	Yes
Hilgert et al. [35]	10	9	69	10	26	169	7	3.50	1.39–8.82	0.008**	1.36	0.41–4.48	0.61	Yes
Hilgert et al. [35]	11	18	69	24	49	169	12	4.90	2.60–9.23	<0.00001**	1.18	0.47–2.96	0.73	Yes

LTF = Number of restorations lost to follow-up; Vol. = Journal volume; DS = Dataset number; N = Number of restorations evaluated; BSL = Number of restorations at baseline; n = Number of failed restorations; RR = Risk ratio; CI = Confidence interval;

*Difference statistically significant in favour of test group;

** Difference statistically significant in favour of control group.

**Table 6:** Assessment of attrition bias risk–Best-case scenario.

Trial	DS	HVGIC group			Resin group			LTF adjusted effect estimate			Original effect estimate			Bias risk
		LTF	N+LTF teeth	n	LTF	N = BSL	n+LTF	RR	95% CI	p	RR	95% CI	p	
Beiruti et al. [14]	01	26	180	0	19	180	24	0.02	0–0.33	0.006*	0.10	0.01–1.70	0.11	Yes
Beiruti et al. [14]	02	26	180	3	42	180	49	0.06	0.02–0.19	<0.00001*	0.38	0.10–1.16	0.16	Yes
Beiruti et al. [14]	03	37	180	4	57	180	65	0.06	0.02–0.17	<0.00001*	0.43	0.13–1.39	0.16	Yes
Beiruti et al. [14]	04	100	180	1	104	180	110	0.01	0–0.06	<0.00001*	0.16	0.02–1.28	0.08	Yes
Barja-Fidalgo et al. [30]	05	25	46	2	18	46	25	0.08	0.02–0.32	0.0003*	0.38	0.09–1.65	0.20	Yes
Oba et al. [31]	06	35	91	6	35	116	43	0.18	0.08–0.40	<0.0001*	1.08	0.40–2.96	0.87	Yes
Chen et al. [32]	07	35	450	7	26	478	31	0.24	0.11–0.54	0.0006*	1.52	0.49–4.77	0.47	Yes
Zhang et al. [33]	08	105	450	9	82	478	96	0.10	0.05–0.19	<0.00001*	0.74	0.32–1.68	0.47	Yes
Liu et al.[26]	09	15	194	13	11	189	18	0.70	0.35–1.40	0.31	1.84	0.75–4.52	0.18	No
Hilgert et al. [35]	10	9	69	4	26	169	33	0.30	0.11–0.81	0.02*	1.36	0.41–4.48	0.61	Yes
Hilgert et al. [35]	11	18	69	6	49	169	61	0.24	0.11–0.53	0.0004*	1.18	0.47–2.96	0.73	Yes

LTF = Number of restorations lost to follow-up; Vol. = Journal volume; DS = Dataset number; N = Number of restorations evaluated; BSL = Number of restorations at baseline; n = Number of failed restorations; RR = Risk ratio; CI = Confidence interval;

*Difference statistically significant in favour of test group;

** Difference statistically significant in favour of control group.

The risk for publication bias could not be assessed due to insufficient data (N < 10) per time frame (24-, 36-, 48- and 60-month follow-up periods)

## Discussion

### Limitations of the Systematic Review Method

The aim of this systematic review was to answer the question as to whether, in patients with fully erupted permanent molar teeth, high-viscosity glass-ionomer based fissure sealants are less effective in protecting against carious lesions in occlusal pits and fissures than resin-based fissure sealants. During this systematic review, non-English publications were excluded. It has been shown that the inclusion of non-English trials has little effect on summary treatment effect estimates and thus can be assumed as confirmatory of trial results published in English [36,37]. Language restricted meta-analyses, compared to language inclusive meta-analyses, do not differ in their effect size estimates (ROR 50.98; 95% CI: 0.81–1.17) [37]. Furthermore, treatment effect estimates from non-English studies are shown in some cases to be 16% more beneficial (Ratio of estimates 0.84; 95% CI: 0.74–0.97; p = 0.011) than that of results published in English [36] and thus may introduce some level of overestimation into meta-analysis results. Thus excluding non-English trials from the systematic review may not have lead to biased results but may have resulted in more conservative treatment effects instead.

Further possible limitations of the applied systematic review methodology include the pooling of datasets from parallel group trials with that from split-mouth studies, the remaining clinical heterogeneity of pooled datasets and lack of publication bias assessment. While it has been reported that data from split-mouth studies require a different statistical analysis than that from parallel group trials [38], empirical evidence suggests that the differences in effect size estimates, including the 95% confidence intervals, between the two study types are minor [39]. For this reason the effect of such methodological heterogeneity on the pooled analysis result was assumed as minor and thus split-mouth data (DS 06 and 09) was pooled in two meta-analyses during this systematic review. The results of the dataset DS 06 and 09 versus DS 02 and 07 for meta-analysis 1 and 2, respectively, confirm the prior assumption of no significant differences between effect size estimates from the two study designs (Fig 2). A number of differences in the treatment settings, treatment procedures, applied sealant materials, tooth selection criteria or trial evaluation have been observed (Tables 1 and 3). In principle, these differences, such as e.g. the presence of fluoride in some resin-based sealant materials and not in others or the differences in applied sealant brands, may have contributed to some level of clinical in-between-trial heterogeneity. However, they do not seem to have had any major impact on the overall heterogeneity as the I^2^ = 0% for the 48- and 60- months follow-up period and 24.2% to 2.0% for 24- and 36-months follow-up period suggest (Fig 2). In line with Cochrane Collaboration recommendation, the measured heterogeneity may thus suggest only moderate statistical heterogeneity without any large effect on the meta-analysis results.

Datasets at 24- and 36- month follow-up periods presented high I^2^- upper confidence levels of 88.2 and 89.8%, respectively. However, as the lower confidence values were both zero, the resulting extremely wide I^2^- confidence intervals may be ascribed to the existing low number of events (Fig 2) rather than to a high statistical in-between-datasets heterogeneity. Because a high overlap of the confidence intervals of the individual dataset results could be observed (Fig 2) suggesting low statistical heterogeneity, it was decided to pool the data for the 24- and 36- month follow-up periods, despite the high I^2^- upper confidence levels.

### Assessment of Trial Validity/Bias Risk

The results of all of the accepted trials appear to be of high selection-, detection/performance- and attrition bias risk. All trials failed to report on evidence of successful sequence allocation and allocation concealment results and most on necessary details about how allocation concealment was attempted (Table 4; Section B in S2 File: Verbatim quotes). For these reasons, none of the accepted trials have provided any guarantee that each trial subject had an equal chance of being allocated to either treatment group. Thus, the internal validity of the systematic results needs to be regarded as low, due to selection bias risk. In addition, none of the trials used the patient as both, the unit of randomisation and the unit of analysis (S2 File / Section 2B), which means that trial results are not based on an even distribution of patient characteristics between both intervention groups. Trials used the sealed tooth as unit of analysis, while randomizing the patient [14,30,32,33] and in one instance the school [32]. This will have let to clustering of data in individual patients without statistical correction in most cases, thus affecting the precision of the effect estimates by artificially narrowing its confidence intervals.

Owing to visible differences between the sealant materials, e.g. the surface of resin-based sealants being of more smooth appearance than that of HVGIC sealants, successful patient and operator blinding appeared not to be possible from the onset in all accepted trials. Therefore, allocation to either group was visible to patients, operators and evaluators and the risk of performance- and detection bias need to be regarded as high. Potential knowledge of superiority claims, e.g. dental association’s statements in favor of resin-based sealants above that of HVGIC [40], may or may not have affected oral hygiene behavior of patients and the quality of placing the sealants by operators (performance bias risk), as well as the application of different rigor by evaluators in their assessment of the different treatment groups (detection bias risk). A potential lack of adequate and successful randomisation of subjects may have resulted in an unequal distribution of confounding factors that may have influenced the results. Such factors are baseline caries experience, caries activity, level of exposure to external fluoride sources, differences in population based caries risk (Table 1) and differences in oral hygiene behavior and these factors were not all adequately reported in the accepted trials.

Based on the quantitative assessment (Tables 5 and 6), attrition bias risk may be regarded as high for all 11 datasets. Due to the number of subjects reported in all trials lost to-follow-up, the calculated results for either of the two extreme scenarios differed significantly from the established trial results. While the latter indicated no statistically significant difference in the failure rate between both types of sealants, the results of all datasets in the assumed “worst-case” scenario indicated in favor (p<0.05) of resin-based sealants while 10 out of 11 dataset results in the assumed “best-case” scenario indicated in favor (p<0.05) of HVGIC-based sealants. Since the true clinical outcomes of sealed teeth in subjects that were lost to follow-up cannot be known, the presented trial results have to be interpreted with caution as they do not appear to be sufficiently robust against doubts that any inclusion of the results from lost subjects may have yielded different effect estimates for datasets and meta-analysis results.

### Analysis Results

Based on the conducted analyses, all datasets showed no statistically significant differences in effect sizes per dataset. When datasets were pooled during meta-analysis the lack of statistically significant differences in the carious rate of sealed teeth was confirmed for follow-up periods up to 48 months (Fig 2). A borderline significance (RR 0.29, 95% CI: 0.09–0.95; p = 0.04) in favour of HVGIC was established after the period of 60 months. This result suggests that teeth sealed with HVGIC have a 71% lower chance of being affected by dental carious lesions than if they were sealed with resin composite. The consequent illustrative risk shows that 4 out of 100 teeth sealed with HVGIC were carious compared to 13 out of 100 teeth sealed with resin composite. In answer to the review question, the currently available evidence suggest that in patients with fully erupted permanent molar teeth, HVGIC based fissure sealants appear not less effective to protect against dental carious lesions in occlusal pits and fissures than resin-based fissure sealants.

The established indicative evidence of a possibly superior caries-preventive effect of HVGIC above that of resin-based sealants remains poor and requires corroboration through future research. In addition, high risk of selection-, performance/detection- and attrition bias (Tables 4–6) questions whether the currently available evidence can be regarded as valid. So far, the current clinical evidence appears to suggest that:

1. Inferiority claims against HVGIC in comparison to resin-based sealants as current gold-standard are not supported;
2. A similar caries-preventive efficacy of HVGIC and resin-based sealants after a period of 48 months in permanent molar teeth appears to exist but remains challenged by high bias risk;
3. The evidence concerning a possible superiority of HVGIC above resin-based sealants after 60 months is poor (even if the high bias risk is disregarded) due to imprecision and requires corroboration through future research.

### Recommendations for Further Research

The available evidence remains limited by bias risk. Future randomised control trials using parallel group study design with sufficiently high number of subjects per intervention group are needed. Such trials should apply adequate methods for randomised subject allocation and allocation concealment. In addition, future trials should include the Berger-Exner test [41] in order to investigate any possible inclusion of third order selection bias during the trial.

## Conclusions

The currently available evidence does not support the claim that in patients with fully erupted permanent molar teeth, HVGIC based fissure sealants are less effective to protect against dental carious lesions in occlusal pits and fissures than resin-based fissure sealants. However, the bias risk is high in all identified trials and challenges the validity of the current results. The evidence in support of similar caries-preventive efficacy of HVGIC and resin-based sealants after a period of 48 months and any possible superiority of HVGIC above resin-based sealants after 60 months remains poor and requires corroboration through future research.

## Supporting Information

S1 File Systematic literature search / Internal validity criteria. (DOC)

S2 File Data extracted from trial reports. (XLS)

S1 PRISMA 2009 Checklist (DOC)

S1 PRISMA 2009 Flow Diagram (DOC)
