# Supplementary material for: Caries-Preventive Effect of High-Viscosity Glass Ionomer and Resin-Based Fissure Sealants on Permanent Teeth: A Systematic Review of Clinical Trials
Source: PLoS One. 2016 Jan 22;11(1):e0146512. doi: 10.1371/journal.pone.0146512 (PMC4723148; doi:10.1371/journal.pone.0146512)
Supplement: S1 File — (DOC) [file pone.0146512.s001.doc]

**Caries-preventive effect of high-viscosity glass ionomer and resin-based fissure sealants on permanent teeth: a systematic review of clinical trials**

Steffen Mickenautsch*, Veerasamy Yengopal*

*SYSTEM Initiative / Department of Community Dentistry, Faculty of Health Sciences, University of the Witwatersrand, 7 York Rd., Parktown/Johannesburg 2193, South Africa.

**Additional file S1 – Section A. Systematic literature search**

| Search term number | Electronic database | Number of Citations found |
| --- | --- | --- |
| BMC search strategy: 16.01.2015  Online: http://www.biomedcentral.com/search/boolean | | |
| [1] | fissure sealant AND glass ionomer AND resin | 12 |
| **Articles included** | | **0** |
| CENTRAL search strategy: 16.01.2015  Online: <http://www.thecochranelibrary.com/view/0/index.html> | | |
| [1] | fissure sealant AND glass ionomer AND resin | 74 |
| **Articles included** | | **0** |
| DOAJ search strategy: 16.01.2015  Online: [http://www.doaj.org](http://www.doaj.org/) | | |
| [1] | fissure sealant AND glass ionomer AND resin | 11 |
| **Articles included** | | **0** |
| GoogleScholar search strategy: 16.01.2015  Online: <http://scholar.google.co.za/> | | |
| [1] | fissure sealant AND glass ionomer AND resin | About 3610 results |
| **Articles included** | | **0** |
| IndMed search strategy: 16.01.2015  Online: <http://indmed.nic.in/> | | |
| [1] | fissure sealant AND glass ionomer AND resin | 0 |
| **Articles included** | | **0** |
| OpenSIGLE search strategy: 16.01.2015  Online: <http://opensigle.inist.fr/> | | |
| [1] | fissure sealant AND glass ionomer AND resin | 0 |
| **Articles included** | | **0** |
| PubMed search strategy: 16.01.2015  Online: [http://www.pubmed.org](http://www.pubmed.org/) | | |
| [1]  [2] | fissure sealant AND glass ionomer AND resin  (GIC sealant* OR glass ionomer cement sealant) AND (caries OR tooth decay) | 179  139 |
| **Articles included** | | **7*** |
| SABINET search strategy: 16.01.2015  Online: <http://www.sabinet.co.za/> | | |
| [1] | fissure sealant AND glass ionomer AND resin |  |
| **Articles included** | | **0** |
| **Reference check of included trial reports** | | **0** |
| **Total articles/documents included** | | **7*** |

Duplications of found citations are excluded.

*Seven trial reports from six trials.

**Additional file S1 – Section B. Internal validity criteria**

1. Assessment criteria for selection bias risk

| Criteria | | Score | | | | |
| --- | --- | --- | --- | --- | --- | --- |
|  |  | 0 | D | C | B | A |
| Adequate random sequence generation method reported^1^ | | N | Y | Y | Y | Y |
| Method of concealing the generated random sequence is reported that is adequate to: | Prevent its direct observation^2^ | N | N | Y | Y | Y |
|  | Prevent its correct prediction^3^ | N | N | N | Y | Y |
| Evidence is given in some form of statistical test result that indicates the allocated random sequence was adhered to throughout the trial^4^ | | N | N | N | N | Y |
| Conclusion | | NA | | | | A |

A = Adequate randomisation; NA = Not adequate randomisation; N = Information not provided in the trial report; Y = Information provided in the trial report.

^1^ The following methods are considered as inadequate: cluster randomisation, fixed block randomisation with block size 2, minimization, alternation, randomisation of teeth, use of date of birth or patient record number, “quasi”-randomisation, split-mouth, generation of the random sequence before patient recruitment.

^2^ Central randomisation or sequence allocation by other than the operator(s) who apply the allocated intervention and who informs the operator(s) which (test- or control) intervention has been allocated to a particular patient only at moment of start of intervention (e.g. by phone), are considered to be adequate.

^3^ Use of the Maximal randomisation procedure is considered to be adequate.

^1-3^ Fulfilment of these criteria indicates adequate attempt of effective randomisation but not that the attempt was indeed successful.

^4^ Any statistical test that includes the use of the Reverse Propensity Score (RPS) is considered to be adequate. Fulfilment of this criterion indicates that the attempt of effective randomisation was indeed successful, i.e. sufficient proof of low selection bias risk.

# .2. - Assessment criteria for detection- and performance bias risk

| Criteria | Score | | | |
| --- | --- | --- | --- | --- |
|  | 0 | C | B | A |
| Adequate method of masking/blinding of patients and clinicians and evaluators reported^1^ | N | Y | Y | Y |
| No reasons for doubt discerned from the report text that masking/blinding was not successful | N | N | Y | Y |
| Evidence is given in some form of applied test* result that shows the masking/blinding was successful throughout the trial^2^ | N | N | N | Y |
| Conclusion | NA | | | A |

A = Adequate masking/blinding; NA = Not adequate masking/blinding; N = Information not provided in the trial report;

Y = Information provided in the trial report.

^1^ Fulfilment of this criterion indicates adequate attempt of effective masking/blinding of effective masking/blinding but not that the attempt was indeed successful.

^2^ Fulfilment of this criterion indicates that the attempt of effective masking/blinding was indeed successful, i.e. proof of lack of detection/performance bias

***** E.g. Application of a questionnaire during the trial that assesses beliefs, as to which test- or control intervention was given/received to/by a particular trial participant, followed by statistical comparison of the questionnaire results with the true allocation sequence. A non-significant result (alpha set at 5%) should sufficiently indicate successful attempt of masking/blinding and subsequent low detection- and performance bias risk.
