# Supplementary material for: Caries-Preventive Effect of High-Viscosity Glass Ionomer and Resin-Based Fissure Sealants on Permanent Teeth: A Systematic Review of Clinical Trials
Source: PLoS One. 2016 Jan 22;11(1):e0146512. doi: 10.1371/journal.pone.0146512 (PMC4723148; doi:10.1371/journal.pone.0146512)
Supplement: S1 PRISMA 2009 Flow Diagram — (DOC) [file pone.0146512.s004.doc]

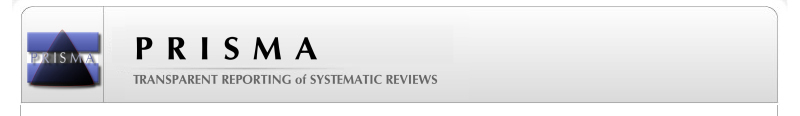
**PRISMA 2009 Flow Diagram**

**Screening**

**Included**

**Eligibility**

**Identification**

Records identified through database searching
(n = 4 025)

Additional records identified through other sources
(n = 1)

Records after duplicates and non-relevant reports removed (n = 4 019)

Records screened
(n = 7)

Records excluded
(n = 1)

Records accepted

(n = 6)

Datasets extracted and included in analysis
(n = 11)
